# Active nudging towards digital well-being: reducing excessive screen time on mobile phones and potential improvement for sleep quality

**DOI:** 10.3389/fpsyt.2025.1602997

**Published:** 2025-07-17

**Authors:** Thao Hanh Vu, Marco Tagliabue

**Affiliations:** Department of Behavioural Sciences, OsloMet - Oslo Metropolitan University, Oslo, Norway

**Keywords:** screen time, digital addiction, digital nudge, sleep quality, smartphone

## Abstract

**Introduction:**

Our society’s reliance on smartphones is a growing phenomenon. Misuse or overuse of smartphones has been associated with negative effects on physical health and psychological functioning, including reduced quality of sleep when used before bedtime. Increasingly, digital users are becoming more aware of how smartphone use impacts their productivity and well-being. Consequently, several digital detox interventions incorporating digital nudges have been introduced to help users reduce their smartphone usage. Digital nudges are freedom-preserving behavior-altering mechanisms that utilize user-interface design.

**Methods:**

In this exploratory study, we examine the effectiveness of a digital nudge—in the form of tracked screen time—as a behavioral intervention to mitigate excessive smartphone use. Secondarily, we explore the potential relationship between screen time and sleep quality. A within-group experimental design, using a randomized controlled trial with a sample of 17 participants, was conducted over 7 days to compare the effectiveness of a tracking-only condition with an active digital nudge condition.

**Results:**

No significant evidence was found to support the impact of the active digital nudge on reducing screen time (primary outcome). There was a direct correlation between screen time reduction and improved sleep quality (secondary outcome), along with a significant effect of reduced frequency of sleep delay in the active nudge condition (*p* = 0.026).

**Discussion:**

Nonetheless, the findings of this study contribute to our understanding of the mechanisms underlying digital nudges and offer valuable insights into how their effectiveness can be improved and optimized from a behavior-analytic perspective.

## Introduction

Since the introduction of the first mobile radio by Bell Laboratories in 1947 in the USA, to the development of cellular phones capable of transmitting and receiving across various radio wave frequencies by the late 1900s, mobile phone technology has evolved rapidly (1). With an expanded range of functionalities, mobile phones have become an integral part of our daily lives, for good and for bad. Smartphone use may offer short-term benefits to adolescents’ well-being, particularly when it involves active social interactions such as instant messaging or social media engagement. These forms of communication can foster and gratify a sense of connection among peers, or with like-minded individuals whom adolescents may not frequently encounter in offline settings (2, 3 as cited in 4). In addition to benefits in strengthening both social communication and work-related cooperation, the use of wearable devices also offers health advantages, including improved physical activity and reduced sedentary behavior (see 5, for a review). Marciano et al. (4) also reported small positive effects on well-being, which may reflect the fulfillment of social needs that adolescents gain from smartphone use, such as emotional expression, enjoyment, and stress-coping tactics. This may suggest a reinforcing loop that contributes to online addictive behaviors due to the emotionally and socially rewarding nature of these activities (6, as cited in 4).

Excessive use of digital devices in general—and mobile phones in particular—has been reported to have detrimental influences on physical functioning and psychological well-being, even before technology addiction was officially recognized as a global issue by the World Health Organization (7, 8). For example, Martínez-Larrínaga et al. (9) examined the relationship between screen time and sleep quality in primary school students and found a significant correlation, which may represent a risk factor extending into adolescence and adulthood.

Psychological well-being is a complex, positive mental state that plays a central role in overall mental health. Tang et al. (10) defined it as “including hedonic (enjoyment, pleasure) and eudaimonic (meaning, fulfillment) happiness, as well as resilience (coping, emotion regulation, healthy problem solving)”. In this study, we focus on the third component of this definition—resilience—as it may be functionally influenced by changes in smartphone use.

According to Peraman and Parasuraman (11), excessive smartphone use is associated with various physical health problems, including a high prevalence of musculoskeletal pain in the wrist, back, or neck—ranging from 8% to 89% (12)—as well as headache, blurred vision, eye strain, and other ocular disorders (13, 14). Beyond these physical effects, the immediate accessibility of digital interactions through smartphones may also lead to a range of sociopsychological issues. These issues include reduced self-confidence, emotional instability, delusions, depression, or nomophobia—a type of anxiety disorder characterized by extreme anxiety or insecurity when disconnected from mobile phone connectivity (15, as cited in 16). Both physical discomfort and psychological issues may contribute to sleep dysregulation at night and daytime dysfunction the next day, as sleep is a fundamental biological process that plays a critical role in regulating mood and emotional functioning (17). This can create a vicious cycle, intensifying symptoms of depression and anxiety (13, 18).

Several studies have investigated these relationships and found that insufficient sleep, poor sleep quality, subjective insomnia, and bedtime procrastination are associated with excessive screen time on mobile phones, especially before bedtime, among adolescents and young adults (17, 19–22). For example, bedtime procrastination has been found to be a mediator between problematic smartphone use and sleep quality among adolescents (aged 13–18) in Turkey (23), and between smartphone addition and sleep quality among university students in China (24), including during the COVID-19 pandemic (25). The context of the pandemic inspired exploratory research on the effects of gender and age in relation to the use of technological devices during teleworking on sleep problems (26). In another study, Figuereido and Kulari (27) examined the role of sleep preferences and daytime chronotype in academic achievement among university students, finding that the morningness type became the most preferred after the pandemic.

Sleep duration may be reduced, as time spent in bed does not equal actual time spent sleeping, and sleep onset latency tends to be longer (28, 29; as cited by 30). The emission of short-wavelength light between 446 and 480 nm (Hannibal et al., as cited in 31), along with electromagnetic fields from mobile device screens, has been reported to delay the melatonin secretion process and disrupt normal circadian rhythms (22, 32; as cited in 21), leading to poor sleep quality and decreased sleep efficiency. Short-wavelength light information is transmitted via the retinohypothalamic tract to the suprachiasmatic nuclei (SCN) of the hypothalamus, which regulate the circadian rhythm and signal the pineal gland, responsible for melatonin production. Late-evening exposure to short-wavelength blue light reduces melatonin levels more strongly than exposure to longer wavelengths or no light 2 h before sleep, suppressing the onset of melatonin secretion and weakening sleep pressure (33–35; as cited in 31).

According to a report by the Norwegian Consumer Council (36), most technology companies, such as Google, Facebook, and Amazon, design their platforms to monetize user data by exploiting their psychological vulnerabilities and maximizing engagement for financial gain. For example, Facebook uses red-colored notifications to trigger a sense of urgency and provides an endless news feed that facilitates an infinite and mindless scrolling experience (37, as cited in Kozyreva, 38). This design approach is based on the Fogg Behavioral Model, which posits that sufficient motivation, necessary competence, and proper triggers are essential for a person to perform a target behavior (39). Fogg (39) emphasized the use of predesigned prompts that align with digital users’ abilities and motivation in order to effectively persuade them toward the desired behavior.

Eyal (40) further introduced the Hook model, which has been widely adopted by technology companies in the development of social media platforms. This model begins with a trigger that prompts an action leading to a reward, thereby initiating a continuous cycle of user investment in anticipation of future rewards. This investment increases the likelihood that users will continue responding to triggers to gain social rewards (e.g., visibility, competence, reputation). The Hook model describes an addictive feedback loop with a high return on investment, grounded in behavior-analytic principles, to build user habits (40). Digital platforms apply these principles to influence user behavior by designing algorithmic systems based on past activity, microtargeting individuals with customized advertisements, and reinforcing both mindless consumption and habitual checking behaviors (40). As a result of these compulsive and addictive behavior patterns, digital users may unknowingly develop a dysfunctional lifestyle.

Problematic Internet use has emerged as a growing social concern, with several studies reporting a negative correlation between it and resilience. In a systematic review and meta-analysis on the topic, Hidalgo-Fuentes et al. (41) found strong evidence of a weak-to-moderate relationship between these variables. One of the studies included in their analysis further revealed the significant negative relationship between problematic Internet use and resilience also extended to affect levels of happiness and dispositional hope (42).

### Digital nudging and its behavioral mechanisms

Thaler and Sunstein (43) defined nudging as “any aspect of the choice architecture that alters people’s behavior in a predictable way without forbidding any options or significantly changing their economic incentives”. According to Sunstein (44), choice architecture refers to the inherent features of the physical environment and social context in which choice behavior occurs. Architecture inevitably influences behavior, even when it is not deliberately designed to do so, because choices are always made within a structured order. The behavioral economics concept of “choice architecture” is considered a libertarian paternalistic approach. It is paternalistic because nudges are designed to modify behavior with clear intentions and predictable outcomes. It is libertarian because individuals are still deemed the best judges of their own choices. In a nudging intervention, all choice options remain available and unaltered, with no manipulation of incentives or imposition of sanctions on undesired behaviors (43). Hansen (45) stated that a nudge should function independently of any sanctions related to time, trouble, or social exclusion, of economically beneficial choice alternatives, and of “the provision of factual information and rational argumentation”. To qualify as a behavior-modification procedure, a nudge must demonstrably influence behavior by exploiting existing cognitive limitations, biases, routines, habits, and social dynamics that prevent individuals from making rational choices aligned with their own preferences. By modifying the choice environment, nudges aim to either stimulate or counteract the effects of heuristics and biases to influence behavior without coercion (45). From a behavior-analytic perspective, choice architecture is described as “a form of arranging individual behavioral contingencies” (46). Choice architecture, or nudging, seeks to change behavior by influencing the natural and social environment, aligning with the principle of bringing behavior under the control of reinforcement contingencies (47). However, nudging focuses more on predicting behavior than on controlling it, and the choice architecture process does not involve the arrangement of reinforcers or punishers. Therefore, nudging cannot be explained using a three-term contingency, in which a response is evoked by an antecedent event, followed by a consequence that increases or decreases the likelihood of that behavior depending on its contingency (48). Andersen and Dechsling (49) suggested that nudging can be categorized as a behavioral analytic tool under the concept of stimulus control. According to Cooper et al. (50), stimulus control results from a history of differential reinforcement: the frequency of a behavior changes in the presence of specific stimuli due to prior learning experiences with similar stimuli of the same class. Heuristics and autonomous behavior function through the process of stimulus generalization, wherein a reinforcement history associated with specific stimuli generalizes to other stimuli sharing similar characteristics (51, as cited in 49). This mechanism can be applied in nudging techniques such as “default options” and “salience”. When implemented in digital environments through “the use of user-interface design elements to guide people’s behavior” (52, p. 433), they are considered “digital nudges”. Given the dramatic acceleration of technological development, the effectiveness of this subset of nudging can be further enhanced through the integration of algorithms, dark nudges (e.g., those undisclosed to users or ethically questionable), and real-time data (53). For example, previous applications of nudging to promote healthy sleep have include sending personalized “coaching” messages (54) and a implementing a multifaceted approach, such as modifying electronic health record schedules, providing reminders, and offering education, which led to fewer unnecessary overnight interventions (55).

Zimmermann and Sobolev (56) conducted a study to assess the effectiveness of digital nudges in reducing screen time. They used a variety of digital nudges, including setting a default schedule to block all apps on mobile phones (the Downtime feature in iOS Screen Time), sending prompts to limit usage on specific apps (App Limits feature), and displaying daily screen time usage (tracked through the Screen Time app). In their study, Zimmermann and Sobolev (56) combined the Downtime and App Limits features as active nudges, used Grayscale Mode as a passive nudge, and included a tracking-only control condition to examine whether these digital nudges influenced screen time. The passive nudge led to an immediate and substantial reduction in objectively measured screen time compared to the control group. Contrary to participants’ expectations that conscious, self-regulated habit formation would be the most effective approach, the active nudge resulted in a more gradual and smaller reduction in screen time. In contrast, participants in the control group, who only tracked their usage, showed no reduction in screen time.

Caraban et al. (57) demonstrated that, over the past decade, digital nudges have been effective in shaping users’ interactions with technology, as recognized by both academics and practitioners. As defined by Weinmann et al. (52), digital nudges are nudges delivered through digital technologies to influence people’s decisions or behaviors within digital contexts. For example, Bergram et al. (58) incorporated digital nudges into the design of a privacy dialog box aimed at interrupting mindless activity and simplifying the task of reading terms and privacy policies, thereby increasing users’ awareness of data privacy online. In studies specifically targeting screen time or mobile phone usage, Purohit et al. (59) tested the use of digital detox apps to examine whether users could reduce their use of social media. However, many participants were reluctant to adopt this approach, expressing concerns about the risk of personal data leakage through third-party applications that collect behavioral patterns (57). Studies on digital nudges have also addressed potential issues, such as the risk of applying a nudge too strongly, which can create friction and reduce usability. For example, in their vibration-based intervention, researchers at Cornell Tech incorporated nudging principles and negative reinforcement strategies to reduce social media usage (60). Each time a user exceeded their allotted daily Facebook usage, the intervention delivered subtle but repetitive vibrations as a digital nudge. While the design effectively reduced Facebook use, its removal left many participants feeling upset, dissatisfied with their online experience, and frustrated by the digital nudge. According to Caraban et al. (57), one possible explanation for this lack of sustainability is that participants experienced a perceived infringement on their autonomy due to the friction introduced by the nudging design. To similarly curb mindless, compulsive behavior on digital platforms, Wang et al. (61) created a Chrome plugin that introduced a 10-s delay before uploading a Facebook post, encouraging users to review the content more carefully. Although the timer could be ignored, they found that many users modified or discarded their posts during the delay period. However, Wang et al. (61) also found that many participants were annoyed and perceived the nudge as time-consuming. To avoid becoming a form of manipulation, nudges should always allow for easy opt-out options. In addition, interventions must remain transparent, with users’ well-being consistently taken into consideration (62). A nudge should clearly reveal its purpose so that users can reasonably understand “the intention behind it, as well as the means by which behavioral change is pursued, could reasonably be expected … as a result of the intervention” (63). In agreement with Thaler and Sunstein’s (43) suggestion for a more proactive approach in certain cases, Purohit et al. (64) proposed that digital users should actively participate in the creation of digital detox nudges. This not only helps preserve personal autonomy but also addresses data privacy concerns and reduces usability risks that may compromise the effectiveness of the intervention.

As Zimmermann and Sobolev’s (56) study found no immediate causal impact of screen time reduction on subjective well-being or academic performance, findings that directly contradict prior research linking excessive screen time to sleep disturbances, the present author conducted a modified replication of Zimmermann and Sobolev’s (56) study. This replication focused on two key research questions: (a) assessing the effectiveness of implementing both App Limits and Downtime as active digital nudges to reduce screen time on mobile phones throughout the day and before bedtime, and (b) examining the potential correlation between reduced screen time and sleep quality. Hence, the purpose of this study is to examine the effectiveness of digital nudging as a behavioral intervention to mitigate compulsive and addictive digital behaviors, specifically targeting the excessive screen time (e.g., engaging in cyber-leisure activities) just before bedtime.

## Method

### Participants and sampling

We recruited a convenience sample with only two selection criteria: (i) participants had to be between 19 and 30 years old, as young adults are not only considered “digital natives”—having been born into and raises with digital technology, and thus more likely to engage in extensive and diverse smartphone use compared to older adults (65, 66; as cited in 67)—but also tend to suffer more from sleep deficiency and poor sleep quality (68); and (ii) participants had to be active iPhone users (as opposed to Android or other operating systems users), due to limitations in standardizing the screen time setup instructions across Android devices, where the function names and interfaces vary by brand, unlike the more consistent setup on iOS.

To ensure that each experimental condition included at least *n* = 10 participants, we aimed to recruit as many participants as possible by broadening the target population to young adults residing in the European Union. All individuals who provided consent (*N* = 22) were enrolled in the study and directed immediately from the consent form to the baseline survey.

The information and consent form were written in English and publicly shared on the first author’s Facebook profile and Facebook groups, accompanied by a brief description of the study’s purpose and participation criteria.

No stratification based on population characteristics was applied. Convenience sampling without stratification poses a threat to the external validity of the results (i.e., higher risk of sampling bias and lack of diversity in the sample—see also 69). Therefore, this study makes no claim of representativeness and should be regarded as exploratory.

Participants were then randomly assigned to one of two experimental conditions and sent setup instructions via the email addresses they provided in the consent form. Simple randomization was carried out using a die roll: participants who rolled even numbers were assigned to the tracking-only control condition, while those who rolled odd numbers were assigned to the active nudge condition. The final sample for the follow-up survey consisted of 17 participants (M age = 25.5 years). Participants who did not confirm completing the setup of the Screen Time function, did not complete the follow-up survey, or failed to follow the instructions and complete the project within 7 days were excluded. Unfortunately, no data on participants’ gender were collected. According to Zimmermann and Sobolev (56), previous research on screen time reduction interventions reported an effect size (Cohen’s *d*) between 0.4 and 0.5. Thus, assuming a sample of 17 participants per condition, our study had sufficient power (62%) to detect an effect in a paired samples *t*-test. This limited statistical power represents a constraint of the study and is discussed further below. In fact, low statistical power reduces the likelihood that statistically significant findings reflect true effects (70).

In their original study, Zimmermann and Sobolev (56) included a control condition, an active nudge condition, and a passive nudge condition. Considering potential limitations anticipated in the present project, which are discussed further in the Discussion, we chose to replicate Zimmermann and Sobolev’s (56) study using only a tracking-only control condition and an active digital nudge condition.

#### Experimental design

Participants who completed the pretest survey were subsequently randomly assigned to one of two conditions: (1) control condition (*n* = 10) and (2) active nudge condition (*n* = 12). The control condition was referred to as the “tracking condition” and the active nudge condition as the “self-commitment condition” in the instructions sent out to participants.

In the control condition, participants were briefly introduced to the Screen Time function and then instructed to track their screen time daily. They were asked to open the function and take a screenshot of their total smartphone usage each night before going to sleep. In the active nudge condition, participants received step-by-step instructions: first, to identify apps they found addictive or detrimental to productivity, and then to set screen time reduction using Downtime and App Limits features within the Screen Time function. Participants were encouraged to use both Downtime and App Limits for optimal results, and the follow-up survey included questions to confirm whether both features had been activated. The instructions explained how to use Downtime to block screen access during specific periods—such as for work, study, or sleep—and how to use App Limits to set daily time restrictions for specific apps or app categories. Clear examples were provided for each condition.

After 7 days of implementing the intervention, participants were asked to complete a posttest survey. The final sample included *n* = 9 in the control condition and *n* = 8 in the active nudge condition. No personal data were collected in either the baseline survey or follow-up survey that could identify or match participants; thus, the entire procedure was anonymous. **Table 1** provides a complete overview of the sample characteristics collected in the baseline survey.

**Table 1:** Sample characteristics of participants before the intervention.

	N	Min.	Max.	M	SD
Focus ability	22	3	5	3.42	0.525
Phone behavior pretest					
Phone dependence	22	2	4	3.14	0.665
Prior use of screen time function (% yes)	22			50	
Estimated daily screen time (minutes)	22	60	480	278.95	101.972
Estimated screen time before bed	22	1	5	3.36	0.953
Estimated number of pickups	22	2	200	29.23	44.049
Estimated percentage of productive screen time (%)	22	5	65	34.91	17.639
Estimated enjoyment towards leisure activities on screen	22	4	10	7.41	1.652
Expected effectiveness					
Receiving detailed information about your individual mobile phone usage	22	1	10	5.82	2.423
Setting yourself time limits for specific apps	22	1	10	5.68	2.607
Setting a specific period of the day to stay away from the mobile phone screen	22	1	10	5.86	2.981

### Materials and procedure

In compliance with personal data protection regulations in Norway, this study was submitted for assessment to the Norwegian Agency for Shared Services in Education and Research (SIKT). The management plan for processing personal information received a positive assessment with reference number 594471. Moreover, because the project collected self-report data on sleep quality, it was sent for preassessment to the Regional Committee for Medical and Health Research Ethics, which concluded that no formal approval was required (reference number 600806). A risk and vulnerability analysis of the management of personal information was performed according to the university’s guidelines and deposited on a public repository system with registration number 20/10901-138.

#### Description of screen time function

The iOS operating system includes the Screen Time function, which gives users a visual representation of their daily and weekly screen usage. Overall screen time data on an iPhone is divided into categories based on app functions, including social networking, productivity, gaming, and reading. Users also have access to data on what apps they use the most frequently, how often they pick up their phone, and how many notifications they get throughout the day. These features enabled participants in the control condition to continue to observe how they were using their mobile devices. In the active nudge condition, two time-management features within the Screen Time function were utilized. The “Downtime” feature allows individuals to schedule designated periods of time when only phone calls and specific apps are accessible, effectively creating a block on screen usage. The “App Limits” feature allows users to set daily time restrictions for categories such as social networking or specific apps that they feel are most addictive. These limits reset every day at midnight, and if a person surpasses their set limit, the app is blocked, accompanied by a notification to ignore limits for 1 min, 15 min, or the whole day. Users have the flexibility to extend or remove the time limits they have set for themselves, so the limits and downtime are not strictly enforced. Due to its liberty-preserving characteristic, the Screen Time function was selected as the digital nudge for this study.

#### Pretest survey

Data collection was handled by questionnaires created with nettskjema.no, a survey solution developed and hosted by the University of Oslo (nettskjema@usit.uio.no). After providing informed consent via the registration form, participants were directed to the pretest survey, which included questions about their self-perceived current smartphone usage and sleep quality. The survey began with a question regarding previous use of Screen Time, followed by several exploratory items assessing their ability to focus, rated on a 5-point Likert scale. Subsequently, respondents were asked to subjectively estimate their total daily screen time on a smartphone in hours and minutes, as well as the usual number of pickups triggered by notifications. They also subjectively estimated the percentage of their productive smartphone usage (0%–100%), rated their enjoyment of leisure activities on a smartphone unrelated to work or study using a 10-point Likert scale, and reported the amount of screen time spent on leisure activities before bedtime. Another exploratory question assessed respondents’ expectations regarding the effectiveness of three techniques for reducing mobile phone screen time: (1) “receiving detailed information about their individual mobile phone usage”, (2) “setting time limits for specific apps”, and (3) “designating a specific period for each day to stay away from mobile phone screen”. Each technique was rated on a 10-point Likert scale. The scoring range used to compare both the 5-point Likert scale and 10-point Likert scale in the pretest survey was calculated according to Wu (71) and is included in the **Appendix**. Data on the overall sleep quality of participants were collected via six measures of sleep quality derived from the Pittsburgh Sleep Quality Index (PSQI) (72). Smartphone dependence was assessed using an 11-item scale adapted from Ward et al. (73).

#### Posttest survey and screen time

After being introduced to control and active nudge conditions, participants were given a posttest survey with questions regarding the project, confirmation of compliance, screen time data, and sleep quality measures after 7 days of the project. All participants were asked to provide objective information on their daily total screen time in the last 7 days of the project in hours and minutes, retrieved from data on the Screen Time app. While proofs in the form of screenshots of screen time usage were not asked for to minimize the complications of the report for participants, this should be required in future research and replications to ensure objective data. The primary dependent variable was the average daily screen time measured in minutes. Subsequently, these data were used to calculate the average daily screen time variable for each participant, enabling a comparison of smartphone usage before and after the nudging intervention.

The posttest survey tailored different questions for each condition. Participants in the control condition rated the extent to which observing their detailed mobile phone usage every day helped them to reduce their screen time in a whole day and before bedtime (1 = not effective at all, 10 = totally effective). Meanwhile, participants in the active nudge condition described how they set up their goals by using Downtime and App Limits, whether they set to use these features every day or only on weekdays, how much the time limits were, and whether they turned on “Block at End of Limit” when using App Limits—which added an extra control factor when applying this type of nudge. Participants in the active nudge condition were later asked how frequently they had broken their own limits and ignored their downtime (1 = never, 5 = about half of the time, 10 = every day), and whether using these features had enabled them to cut back on their screen time before bedtime and during the day (1 = not effective at all, 10 = totally effective).

All participants submitted an estimate of how much time they spent using their smartphones for work-related or productive purposes during the project (ranging from 0% to 100%), how committed they were to lowering their screen time (1 = not much, 10 = absolutely committed), and how much they enjoyed using their phones for recreational purposes during the project (1 = not at all, 10 = very much). Additionally, they ranked how much they thought the Screen Time function had helped them to curb their screen time before bed and during the day (1 = certainly not, 10 = definitely yes). Finally, the identical set of questions from the pretest survey was answered by each participant to gauge their overall quality of sleep. They answered a demographic question regarding their age to conclude the posttest survey.

#### Overall sleep quality

Overall sleep quality is measured based on the PSQI, which is a set of nineteen self-rating questions assessing sleep quality and disturbances (72). We extracted six items from the PSQI to measure overall sleep quality: subjective sleep quality, sleep latency, sleep duration, sleep disturbances, bedtime, and wake-up time. For this study, sleep disturbances were specifically asked for, measured as the frequency of sleep delay due to screen time as leisure activities before bedtime. According to the PSQI (72), overall sleep quality is a composite score measured by first assigning a component score to each question with a range of 0–3 points and then adding the component scores together. This final composite score, with a range of 0–21 points, indicates 0 as having no difficulty in sleep and 21 as having severe difficulties in all areas of sleep.

We extended the range of the sleep quality response options and different scales with the intention of increasing the sensitivity of responses and offering participants more independence to choose exactly what they prefer (74). Subjective sleep quality was measured on a 10-point Likert scale with 1 as absolutely bad sleep and 10 as absolutely good sleep, sleep latency was measured on a 5-point scale (1 = 15 min or less, 5 = more than 2 h), 4-point scale for sleep duration (1 = more than 7 h, 4 = less than 5 h), 7-point scale for bedtime (1 = 9–10 pm, 7 = after 3 am), 6-point scale for wake-up time (1 = 5–6 am, 6 = after 10 am), and 4-point scale for sleep delay frequency (1 = not usually, 4 = every day). Although all components—except for subjective sleep quality—were worded and categorized according to the PSQI, with lower values indicating better sleep patterns and higher values reflecting problematic sleep quality, the scores needed to be recalculated on a 10-point scale to allow comparison of overall sleep quality before and after the intervention.

### Data preparation and analysis

The data were analyzed using IBM SPSS Statistics (Version 27). The subjective sleep quality variable, which was originally contradictorily worded, underwent reverse recoding in the SPSS data file. This recording was done to assign a value of 1 to represent absolutely good sleep and a value of 10 to represent absolutely bad sleep. The mean value for each component of the overall sleep quality was then computed and converted from the component’s original scale to an equivalent value on a 10-point Likert scale. This transformation was employed to ensure that all items were measured on a consistent scale. We derived a composite score by summing the scores from all the component scores to evaluate overall sleep quality.

Paired samples *t*-tests were used to analyze the data before and after the intervention. Independent samples *t*-tests were used to analyze the data between conditions. Spearman’s rank-order correlations were run to examine the relationships between average daily screen time and measures of overall sleep quality before and after intervention.

Within-group comparisons on the primary outcome (i.e., screen time) were performed using nonparametric statistics due to both small sample size and nonnormal distribution of posttest scores (results are reported below). Between-group comparisons on the secondary outcome and the exploratory outcomes were performed using parametric statistics. Although the *t*-test assumes normality of the data, the test seems to be robust to moderate violations^1^ of this assumption, particularly with sufficiently large sample sizes (76). In line with the recommendations of Skaik (77), the *t*-test can be used to analyze samples larger than 15 on the condition that there are no severe outliers, as in our dataset.

For all performed tests, we report whether the test meets the normal distribution assumption by including the results of Shapiro–Wilk tests (primary and exploratory outcomes) or checking the equality of variances through Levene’s test (secondary outcome). Effect size results are consistently reported in the form of Cohen’s *d*, irrespective of their statistical significance, and interpreted based on Cohen (78). This practice is in line with the recommendations of, among others, Sullivan and Feinn (79) and Maher et al. (80) and aims at enhancing transparency of findings and reaching beyond the binary decision departing from the null hypothesis to include a measure of magnitude (81).

## Results

### Primary outcome: screen time

The sample’s mean estimated daily screen time before the intervention was recorded as 278.95 min (SD = 101.97, *N* = 22). Following the intervention, the average daily screen time increased to 312.88 min (SD = 158.69, *N* = 17). Given the small sample size, determining the distribution of the variable screen time was important for choosing an appropriate statistical method. A Shapiro–Wilk test was performed and showed that the distribution of pretest screen time was normally distributed (*W* = 0.965, *p* = 0.733), but the variable posttest screen time departed significantly from normality (*W* = 0.869, *p* = 0.022). Based on this outcome, a non-parametric test was used, and the median with the interquartile range was used to summarize the variable screen time. A Wilcoxon signed-rank test showed that our intervention did not elicit a statistically significant change in screen time reduction (*Z* = − 0.497, *p* = 0.619). Median pretest screen time was 300 min, and posttest screen time was 288 min. We applied the formula *Z*/√*n* to calculate the effect size, which returned a value of 0.012 (i.e., a small effect, according to Cohen’s classification of effect sizes).

Next, we compared the average daily screen time between the nine participants in the control group (M = 299.29, SD = 148.33) and the eight participants who performed the active nudge intervention (M = 328.29, SD = 178.63). We performed a new Shapiro–Wilk test to check that the normality assumption was met. While the results in the tracking-only condition were normally distributed (*W* = 0.922, *p* = 0.407), the results in the active nudge condition were not normally distributed (*W* = 0.814, *p* = 0.040). Thus, we analyzed between-groups differences using a Mann–Whitney *U* test, which did not indicate any significant difference (*U* = 31, *p* = 0.630). The effect size was calculated using the same formula reported above and resulted in 0.117 (i.e., a small effect, according to Cohen’s classification of effect sizes). This result suggests that the active nudge condition, which incorporated both time limits and a downtime schedule, was no more effective in reducing screen time than the control condition, which involved only self-monitoring and observing screen time usage.

**Figure 1:**
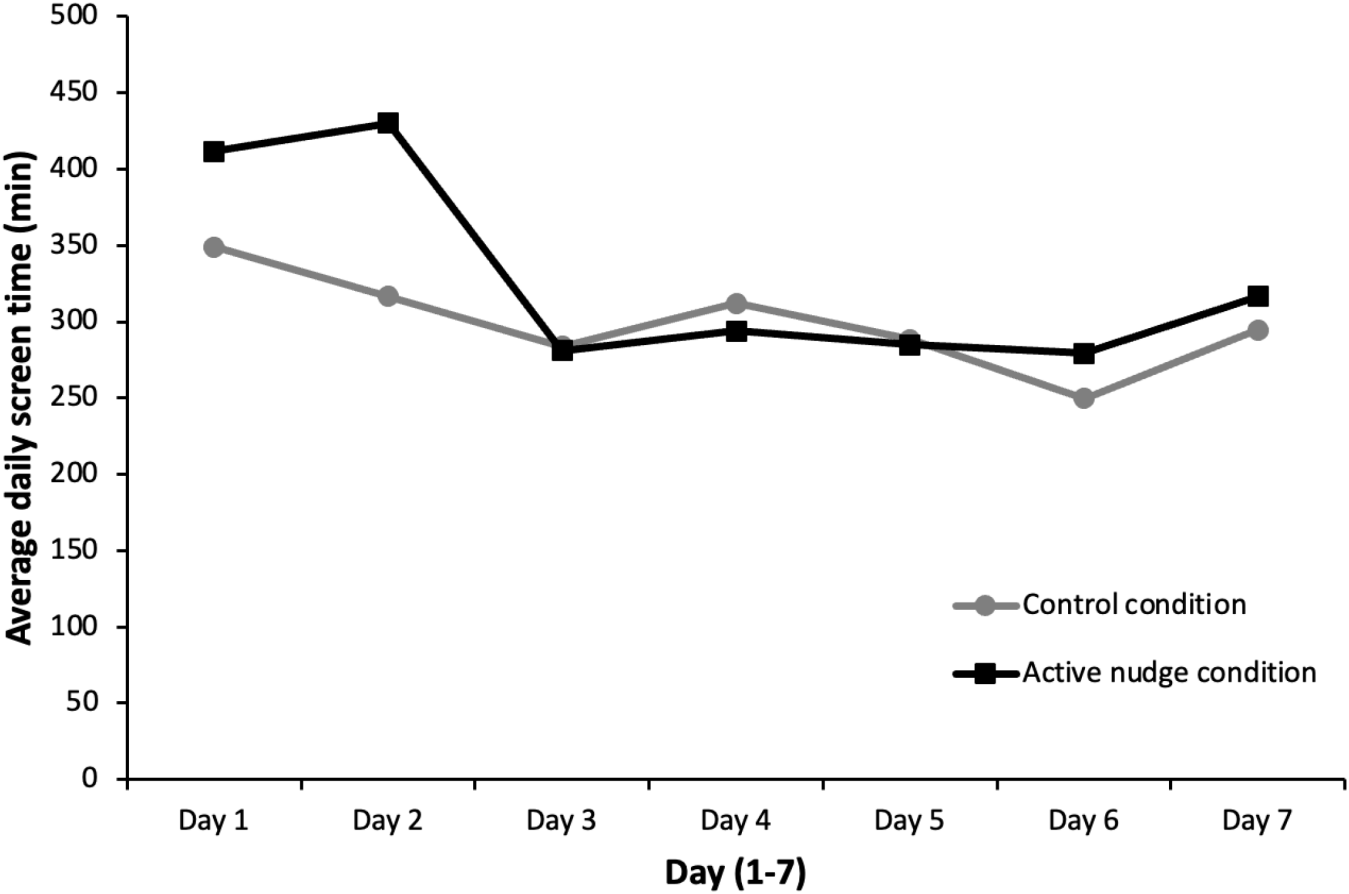
Screen time progression over 7 days. Average daily screen time (in minutes) across 7 days for the control condition group and the active nudge condition group.

To gain an overview of the average screen time progression over a span of 7 days for participants in both conditions, a line graph was employed. Participants’ screen time in the control condition showed minimal variation across the 7 days, while participants with app limits and downtime displayed a substantial reduction in their usage after the first 2 days of intervention. However, the trend witnessed a slight growth on the final day of the project (see **Figure 1** for details). The results of the nonparametric statistical analyses for both the within-group (a) and between-group (b) analyses are included in **Table 2**.

**Table 2:** Results of parametric (a) and non-parametric (b) repeated measures test of the primary outcome (screen time).

(a)								
*Descriptive statistics*								
	N	Mean	Std. deviation	Minimum	Maximum	Percentiles		
						25th	50th (median)	75th
pretest_scrt	22	278,95	101,972	60	480	180,00	300,00	360,00
posttest_scrt	17	312,76	158,788	111	625	180,50	288,00	386,50
*Ranks*								
						N	Mean Rank	Sum of ranks
posttest_scrt - pretest_scrt				Negative Ranks		8^a^	8,25	66,00
				Positive Ranks		9^b^	9,67	87,00
				Ties		0^c^		
				Total		17		

a posttest_scrt < pretest_scrt.

b posttest_scrt > pretest_scrt.

c posttest_scrt = pretest_scrt.

**Table:** 

(b)								
*Descriptive statistics*								
	N	Mean	Std. deviation	Minimum	Maximum	Percentiles		
						25th	50th (median)	75th
average_daily_screentime	17	312,88	158,698	111	625	180,43	288,00	386,50
Experimental condition that partcipant followed	17	1,47	,514	1	2	1,00	1,00	2,00
*Ranks*								
	Experimental condition that partcipant followed					N	Mean rank	Sum of ranks
average_daily_screentime	Tracking-only condition					9	8,44	76,00
	Active nudge condition					8	9,63	77,00
	Total					17		

### Secondary outcome: overall sleep quality

An independent samples t-test was carried out to investigate whether there was any statistically significant difference between control and active nudge conditions for sleep quality variables. The Levene’s test for equality of variances for subjective sleep quality, sleep duration, bedtime, wakeup time, and frequency of sleep delay because of screen time, respectively, had a *p*-value of 0.308, 0.649, 0.803, 0.067, and 0.150. Since these values were greater than 0.05, there were no statistically significant differences in the variances between the two groups of conditions. Equal variances, however, did not meet the assumption in the Levene’s test for the sleep latency variable as the *p*-value was 0.021.

The two-tailed *p*-value for subjective sleep quality, sleep duration, bedtime, and wake-up time measures indicated that there was also no statistically significant difference in the means between the two conditions either, with medium to large effect sizes as measured by Cohen’s *d*, ranging from *d* = 0.62 (medium) for sleep latency, *d* = 0.67 (medium) for usual bedtime, *d* = 0.91 (large) for sleep duration, *d* = 0.96 (large) for wake-up time, and *d* = 1.01 (large) for subjective sleep quality.

Although the variances between the two conditions were likely equal for the frequency of sleep delay because of screen time variable, the results indicated that participants in the active nudge condition (M = 1.38, SD = 0.518) had a significantly lower frequency of sleep delay because of the screen time in comparison with participants in the control condition (M = 2.22, SD = 0.833); *t*(15) = 2.477, two-tailed *p* = 0.026. The effect size was large, with a Cohen’s *d* of 1.20 (see **Table 3** for more details).

**Table 3:** Results for pre- and posttest survey on sleep quality measures and exploratory measures across conditions.

	Pretest	Posttest		
		Control	Active nudge	Test statistic
Subjective sleep quality	6.41 (1.82)	3.67 (1.2)	2.63 (0.92)	*t*(15) = 2.084, *p* = 0.055
Sleep latency	1.91 (1.23)	1.78 (1.39)	1.13 (0.34)	*t*(15) = 1.356, *p* = 0.208
Sleep duration	1.75 (0.54)	1.89 (0.60)	1.38 (0.51)	*t*(15) = 1.877, *p* = 0.080
Usual bedtime	3.32 (1.21)	3.89 (1.26)	3.13 (0.99)	*t*(15) = 1.370, *p* = 0.191
Usual wake-up time	2.68 (1.17)	3.56 (1.23)	2.63 (0.51)	*t*(15) = 1.975, *p* = 0.067
Frequency of sleep delay because of screen time	3.00 (0.873)	2.22 (0.83)	1.38 (0.51)	*t*(15) = 2.477, *p* = 0.026
Percentage of productive screen time (%)	34.91 (17.639)	34.44 (19.558)	53.13 (14.377)	*t*(15) = 2.218, *p* = 0.042
Enjoyment towards leisure activities on screen	7.41 (1.652)	6.78 (0.972)	7.50 (1.690)	*t*(15) = 1.097, *p* = 0.290
Level of commitment to reduce screen time	–	5.67 (2.550)	7.50 (0.756)	*t*(15) = 1.953, *p* = 0.070
Perceived effectiveness of screen time to reduce usage throughout the day	–	6.44 (2.603)	7.38 (2.615)	*t*(15) = 0.734, *p* = 0.474
Perceived effectiveness of screen time to reduce usage before bedtime	–	5.00 (3.279)	9.00 (1.069)	*t*(15) = 3.459, *p* = 0.006

In the pretest data, no correlation was found between average daily screen time and usual bedtime, sleep latency, sleep duration, sleep disturbance, or subjective sleep quality. However, there was a moderate negative and significant correlation between average daily screen time and usual wake-up time, *r_s_* (20) = − 0.43, *p* = 0.043 (i.e., medium to large effect). The posttest data, on the other hand, did not reveal any significant correlation between average daily screen time and any of the measures of overall sleep quality (please see **Table 4** with a complete correlation matrix).

**Table 4:** Nonparametric correlation matrix.

	Average_daily_screentime	Usual bedtime everyday	How long does it take to fall asleep	Usual wake-up time	Hours of actual sleep	Frequency of sleep delay because of screen time	Subjective sleep quality
Spearman’s rho							
average_daily_screentime							
Correlation coefficient	1.000	0.142	0.073	− 0.434^*^	− 0.004	0.262	0.102
Sig. (two-tailed)		0.530	0.745	0.043	0.986	0.239	0.652
*N*	22	22	22	22	21	22	22
Usual bedtime everyday							
Correlation coefficient	0.142	1.000	− 0.132	0.345	0.369	0.190	− 0.161
Sig. (two-tailed)	0.530		0.559	0.116	0.099	0.398	0.475
*N*	22	22	22	22	21	22	22
How long does it take to fall asleep							
Correlation coefficient	0.073	− 0.132	1.000	0.159	0.026	0.574^**^	− 0.568^**^
Sig. (two-tailed)	0.745	0.559		0.481	0.912	0.005	0.006
*N*	22	22	22	22	21	22	22
Usual wake-up time							
Correlation coefficient	− 0.434^*^	0.345	0.159	1.000	0.083	0.247	− 0.483^*^
Sig. (two-tailed)	0.043	0.116	0.481		0.719	0.268	0.023
*N*	22	22	22	22	21	22	22
Hours of actual sleep							
Correlation coefficient	− 0.004	0.369	0.026	0.083	1.000	0.156	− 0.241
Sig. (two-tailed)	0.986	0.099	0.912	0.719		0.500	0.294
*N*	21	21	21	21	21	21	21
Frequency of sleep delay because of screen time							
Correlation coefficient	0.262	0.190	0.574^**^	0.247	0.156	1.000	− 0.336
Sig. (two-tailed)	0.239	0.398	0.005	0.268	0.500		0.127
*N*	22	22	22	22	21	22	22
Subjective sleep quality							
Correlation coefficient	0.102	− 0.161	− 0.568^**^	− 0.483^*^	− 0.241	− 0.336	1.000
Sig. (two-tailed)	0.652	0.475	0.006	0.023	0.294	0.127	
*N*	22	22	22	22	21	22	22

^*^Correlation is significant at the 0.05 level (two-tailed).

^**^Correlation is significant at the 0.01 level (two-tailed).

After being calculated and converted into equivalent values on a 10-point Likert scale, all six component scores were added up to be a final composite score that represents overall sleep quality. The results showed that the composite score in the pretest is 29.6 and 29.2 in the control condition, while it is only 20 in the active nudge condition (on a scale of a maximum of 60 points from six components). The interpretation of this composite score followed PSQI guidelines, where higher scores indicate poorer overall sleep quality and lower scores reflect better sleep quality.

### Exploratory outcomes

#### Effectiveness expectations before the intervention

There were no significant disparities in the perceived effectiveness of the three distinct Screen Time features aimed at reducing screen time. App Limits (M = 5.68, SD = 2.607) and Downtime (M = 5.86, SD = 2.981) were not expected to be more effective than the tracking-only technique (M = 5.82, SD = 2.423), which solely involves self-control observation.

#### Acceptability and perceived effectiveness after the intervention

Approximately half of the participants in the active nudge condition reported surpassing and disregarding their self-set goals for limits and downtime around 50% of the time (M = 4.5, SD = 1.69). Additionally, frequencies indicated that roughly 38% of participants in the active nudge condition found App Limits useful for reducing screen time throughout the day (M = 7.25, SD = 2.816), while half of the participants agreed that App Limits effectively reduced screen time before bedtime (M = 8.38, SD = 1.3). More than 70% of participants in the active nudge condition rated Downtime as moderately to certainly effective and useful for reducing screen time both throughout the day (M = 7.75, SD = 2.375) and before bedtime (M = 8.63, SD = 0.916). Meanwhile, over 40% of participants in the control condition rated that tracking-only feature as moderately ineffective or having no effect on reducing screen time throughout the day (M = 6.33, SD = 2.45).

In general, we assessed whether the perceived efficacy of utilizing the Screen Time function to reduce overall screen time throughout the day was normally distributed using the Shapiro–Wilk test. The results (*W* = 0.942, *p* = 0.602 for the tracking-only condition; *W* = 0.824, *p* = 0.051 for the active nudge condition) indicated that the data were approximately normally distributed. An independent *t*-test revealed no significant difference between the active nudge condition and the control condition (*M*_control = 6.44, *M*_active = 7.38, *t*(15) = 0.734, *p* = 0.474). Cohen’s *d* returned a value of 0.357, indicating a medium effect size.

Next, we tested the normality of the data for the effectiveness of using screen time to reduce screen time before bedtime with a Shapiro–Wilk test, which indicated that this assumption was met (*W* = 0.895, *p* = 0.223 in the tracking-only condition and *W* = 0.860, *p* = 0.120 in the active nudge condition). The results of an independent *t*-test indicated that participants in the active nudge condition indicated a significantly greater perception of the effectiveness of Screen Time in reducing smartphone usage before bedtime compared to participants in the control condition (*M*_control = 5, *M*_active = 9, *t*(9.867) = 3.459, *p* = 0.006). The effect size was large based on Cohen’s *d* = 1.598.

The data for participants’ commitment to reducing screen time met the normality assumption in the tracking-only condition, as indicated by the results of a Shapiro–Wilk test (*W* = 0.948, *p* = 0.666), but failed to do so in the active nudge condition (*W* = 0.724, *p* = 0.004). Notably, there were no significant differences in the level of commitment to reducing screen time between the two conditions (*M*_control = 5.67, *M*_active = 7.5, *t*(9.554) = 2.058, *p* = 0.068)^2^.

#### Productivity and leisure enjoyment

Participants in the active nudge condition demonstrated a significantly greater percentage of productive time during the nudging intervention compared to participants in the control condition (*M*_control = 34.44, *M*_active = 53.13; *t*(15) = 2.218, *p* = 0.042). These data were not normally distributed according to the results of a Shapiro–Wilk test in the tracking-only condition (*W* = 0.781, *p* = 0.012), but they did follow a normal distribution in the active nudge condition (*W* = 0.847, *p* = 0.088). The effect size was large with a Cohen’s *d* of 1.078.

However, the level of enjoyment toward leisure activities did not differ between the two conditions (*M*_control = 6.78, *M*_active = 57.50; *t*(15) = − 1.097, *p* = 0.290). These data were tested for normality and met this assumption through the results of a Shapiro–Wilk test (*W* = 0.903, *p* = 0.273 in the tracking-only condition and *W* = 0.935, *p* = 0.563 in the active nudge condition).

## Discussion

The primary focus of the thesis was to investigate the potential for reducing excessive smartphone usage through the implementation of active digital nudges. Additionally, the study aimed to explore the correlation between screen time and overall sleep quality. This research was conducted as a systematic replication of Zimmermann and Sobolev’s (56) original study, which yielded positive outcomes. Although exploratory due to sampling limitations, the findings of this study suggest that screen time may be influenced by active nudges, albeit to a moderate degree. Results from the independent *t*-test addressing the primary outcome showed no statistically significant effect of active nudges on screen time reduction. However, active digital nudges appeared to contribute to a noticeable decrease in screen time after a few days.

This result is inconsistent with the findings of Zimmermann and Sobolev (56). Although the experimental conditions were similar, the discrepancy may be attributed to the small sample size in our study. Recruitment and follow-up were hindered by the level of engagement required to implement active nudges and reflect on phone usage. According to Sunstein’s (82) explanation of why nudges may fail, a nudge can be ineffective when the perceived cost of rejecting it is minimal and loss aversion is irrelevant. In the context of this thesis, it is possible that default rules such as time limits and prescheduled downtime do not exert sustainable influence on the desired behavior. This lack of influence may arise because individuals do not perceive any tangible negative consequences, either economically or mentally, when they intuitively reject these nudges. The rational evaluation of the costs and benefits associated with ignoring active digital nudges might also be affected by the availability heuristic (82). For instance, individuals may experience inertia during the day, leading them to compulsive and habitual screen checking that gives immediate access to satisfaction rather than engaging in thoughtful reflection on the advantages of reducing screen time. Moreover, Hummel and Maedche (83) posited that the effect of nudges does “not only depend on the nudge itself but also on how it is perceived by an individual”, as many studies indicated that the effectiveness of nudging can be moderated by strong personal preferences (82).

### Active nudges

The active nudges employed in this experiment adhered strictly to nudging principles. They were designed to be simple to opt out of and did not cause any intense friction, allowing participants to continue enjoying their leisure time on screen. Unlike other digital nudging approaches that required participants to install external applications or plugins to activate interventions, this experiment took advantage of built-in features on iPhones as cost-free solutions to address mobile overuse and mitigate the risk of personal data breaches. Nevertheless, it is this soft paternalistic nature of the nudging approach without any form of compliance or coercion that indicates a substantial lack of the control required to be a complete behavioral modification intervention. Hayes and Brownstein (84) stated firmly that the behavior-analytic perspective for the goals of science emphasizes control in the inclusion of prediction. The emphasis on *contro*l is for the demonstration of the direction of influence between variables that are assumed to be functionally related. Strict control of behavior through environmental variables is extremely difficult to achieve under nonexperimental conditions. This point of view has been adopted by most behavior analysts working in the applied branch of science; here, we prefer the more palatable term of *influence.*

The old pattern of phone usage began to gradually recover after a few days, even when the active nudges seemed to take effect. This could be attributed to how frequently the app limits and downtime were broken and ignored by participants. Specifically, 50% of the participants (*N* = 8) admitted to having ignored the limits and downtime half of the time during the 7-day project duration. Further strategies for the implementation of nudges are thus needed to establish the long-term effects of nudging. Simon and Tagliabue (48) suggested a behavior-analytic viewpoint that entails designing a change in the contingency between choices and consequences is necessary to achieve and sustain the desired behavior change when a nudge fails. In fact, no behavior change is maintained without behavior being brought into contact with its reinforcement contingencies. The authors suggest that more adaptive nudging, such as setting up microgoals as reinforcement loops or mood-tracking prompts to offer personalized reminders whenever stress or fatigue from excessive screen time use occurs, might be helpful to improve the practical prevalence of future digital active nudges. For example, an app called “Forest”, which is available for both iOS and Android operating system devices, offers certain features for personalized nudges to concentrate better without smartphone overuse. The app gives users the choice of building their own garden by choosing a certain type of tree or flower to grow during their focus time. Each type of flower requires a different amount of time to grow. For example, tulips only require half an hour to fully grow, while peonies need 1 h to bloom to full size. Users can choose the type of flower depending on how much time they want to dedicate fully to their work, and if they exit the app at any point during the growing period to use other apps, the flower will wither, and they will lose their progress with that flower. Each flower and tree, therefore, functions as a microgoal, and the Forest app also has a Friends mode so that people can add each other and see each other’s progression of focusing on this app. This can be considered a nudge that is adaptive to those who favor the aesthetic of a digital garden with no monetary incentives, easy to opt out, totally transparent, and compliant with nudging principles.

After the active nudge as an independent variable was introduced, there was not much difference in sleep quality component measures, as expected, compared with those in the control condition, except for a significant improvement in the frequency of sleep delay because of screen time. This result was in line with results from previous studies, which found that personal phone overuse was associated with sleep disturbances for young adults (17, 20).

Decreased sleep disturbances also illustrate the positive perceived effectiveness of the nudging intervention in reducing screen time before bedtime from participants in the active nudge condition. Quite surprisingly, further results from Spearman’s rank-order correlation tests did not show any relationship between average screen time and any of the overall sleep quality measures. Sleep quality is a complex construct with many dimensions to it. On the other hand, nudges are relatively simple and targeted interventions and may affect only a limited aspect of sleep quality (e.g., sleep latency or frequency of sleep delay). Thus, more complex behavioral repertoires may benefit from a nudge “plus” initiative, which incorporates elements of reflection to improve its effectiveness and aligns it better with the agent’s autonomy and agency (85). From a behavioral perspective, these nudges are not only featured by acquiring stimulus control but also stimulus generalization: learning to respond to a wider set of stimuli compared to the original setting (86).

Wake-up time was negatively correlated with daily screen time before the intervention; however, after the intervention, there was no evidence that any changes in average daily screen time would harm sleep quality. The overall sleep quality appeared to be better for participants in the active nudge condition and participants before the intervention, as those who were in the tracking-only condition seemed to have more difficulties sleeping. Mixed results of nudging on sleep quality through smartphone use were also shown in the work by Olson et al. (87), with small changes in their participants’ cognition and mood. Moreover, they reported that smartphone use among their participants reverted to preintervention levels 6 weeks after the onset of their nudge-based intervention.

### Study limitations

We referred to our study as *exploratory* due to three main limitations: the small sample size, the self-report nature of the data collection, and the short duration of the intervention.

The first limitation of our study was the small sample size, which reduces the robustness and the generalizability of the findings. Small sample size increases the likelihood of type II errors (false negatives; see 88).

Although we did not find a statistically significant main effect of the active nudge, the small sample size does not pose as great a threat to our conclusions as it would have if a significant effect had been observed. Nevertheless, very small sample sizes undermine both the internal and external validity of the research (89). It is possible that the results were influenced by sampling bias, as participants were primarily recruited from the first author’s network. Moreover, gender information was not collected, and due to the small sample size, no inferences could be made regarding potential gender effects on the influence of the independent variables on any of the dependent measures.

Future research should adopt broader recruitment strategies to increase sample size and power analysis, and to diversify the participant pool by including both iOS and Android users, thereby reducing biases associated with convenience sampling. As Eysenbach (90) noted, “conclusions drawn from a convenience sample are limited and need to be qualified in the Discussion section of a paper”. Accordingly, future studies could follow the Checklist for Reporting Results of Internet E-Surveys (CHERRIES) when designing and administering online questionnaires in the medical field.

The second limitation concerns the self-report nature of data collection, which may threaten the internal validity of our findings. For example, participants were not required to submit screenshot confirmations or other forms of objective verification when reporting their dependent measures. While this approach likely reduced response burden and increased participation, it may also have compromised the accuracy or truthfulness of the data provided. Grayscale mode, used as a passive nudge in the original study, was not included in this study due to its absence in later versions of iOS. Consequently, this replication included only a control condition and an active nudge condition.

The third limitation concerns the duration of our study: it was conducted in a much shorter period (1 week) than the original study of Zimmermann and Sobolev (56), and this may explain the insignificant effect of active nudges in this thesis, as the active nudges were indicated to have a more gradual effect over a span of 3 weeks. A high percentage of participants claimed that they have used the Screen Time function before, and the transparency in the purpose of the study may result in biases from participants’ “perceived expectations of improvement” (50). The prior use of the Screen Time function might be a confounding variable to be controlled for future replication of this study.

The imprecision and inconsistency of scales in measurement tools in this experiment are another possible confounding variable. The data preparation procedure taken to translate the sleep quality component scores into more comprehensive and comparable values, as well as the calculation of the composite score for overall sleep quality, are certainly not the most rigorous assessments, but it was our best attempt to correct for some of the issues with the original PSQI. The original tool gave instructions to calculate a component of sleep quality called “habitual sleep efficiency” as a percentage by dividing the actual hours slept (sleep duration) by the total hours spent in bed, which equals usual wake-up time minus usual bedtime (72). However, as this study followed the original tool to measure sleep duration with values assigned to the Likert-type scale as an approximate range, for example, 1 as more than 7 h and 2 as from 6 to 7 h, the data collected were not precise to the minute for appropriate calculation. After careful consideration, we decided to calculate and compare composite scores of overall sleep quality, a measure that was consistent across participants, even while it contains validity issues. This scoring method was not subject to validation, and future studies should address the lack thereof. When comparing it with other sleep quality assessments, the PSQI showed a high correlation with the Sleep Quality Scale (SQS; 91), and the Sleep Regulatory Questionnaire 92) was introduced to measure subjective sleep regularities.

Sohn et al. (93) emphasized that the duration of smartphone use alone does not indicate addiction but rather reflects an increased likelihood of developing behavioral addiction patterns. Therefore, future research should investigate whether a broader range of smartphone usage patterns is more strongly associated with smartphone addiction and its potential interaction with adverse effects on sleep and other health outcomes. There is a strong demand for a deeper understanding of the underlying mechanisms of smartphone overuse and for the development of effective interventions, both of which are of theoretical and practical significance.

### Directions for future research

Our study represents a systematic replication of Zimmermann and Sobolev (56), with deliberate modifications made to specific elements of the original design. These changes were deemed necessary due to the considerable challenges of replicating the study in natural settings, where numerous uncontrolled variables are prevalent. Furthermore, direct replications, especially intrasubject replication studies, may raise ethical concerns regarding their implementation. Conducting the experiment repeatedly may exacerbate potential damage to the subject by inducing irreversible behavioral changes (94, as cited in 95).

Studies involving humans also require careful consideration of the complexities associated with their “lengthy, varied and unknown histories that might interact in significant ways with experimental procedures” (95), as a single participant may respond differently under even subtle forms of pressure. For instance, participants’ perceptions of what constitutes a good night’s sleep may influence both the perceived effectiveness of a digital nudge aimed at improving sleep quality. In this particular experiment, individual identities could have been matched for analytical purposes by asking participants to provide their email addresses in both the pre- and posttest surveys. However, to minimize the collection of personal information and for ethical considerations, the project only collected names and email addresses in the information and consent form. As a result, participants cannot be identified in either the pre- or posttest survey. Only data collected from the pre- and posttest surveys were transferred from the Excel files generated by Nettskjema, and only numerical data were used for analysis in this study. This procedure was specified in advance during the project evaluation by SIKT and complied with the framework for balancing data openness with participant confidentiality in psychiatric and behavioral research, as proposed by Zhang et al. (96), classifying the data as ranging from moderately sensitive to minimally sensitive.

Further research should also address the diminishing effect of the nudge as participants progressed through exposure to the independent variable (see also 97). From a functional perspective, this may be attributed to the fading of the stimulus control exerted by the change in choice context. In other words, participants may have become so accustomed to the nudge that it no longer influences their behavior. Alternatively, the immediate and gratifying consequences associated with the unwanted behavior may have outweighed the delayed but healthier outcomes promoted by the nudge. This phenomenon is known as temporal discounting, wherein individuals choose smaller-sooner rewards over larger-later ones (98). Therefore, for future interventions to succeed, it is important to design nudges that not only initiate behavioral change but also help maintain it over time, especially in the face of temptations and competing reinforcers—potentially by incorporating elements such as boosts (see 99).

Finally, nudge interventions raise some questions regarding their foundational principles. One such issue is the preservation of individual freedom of choice, which is considered a core criterion for an intervention to qualify as a nudge. A key point of debate is the extent to which nudges implemented by third parties, such as governments or policymakers, can genuinely uphold individual autonomy and freedom of choice (100). Even when the choices offered are not restricted by explicit obstacles, the architecture in which they are presented may still constrain an individual’s autonomy.

Nudges can obscure transparency by obfuscating choices at the moment of decision-making, thereby impeding freedom of choice (101, as cited in 100). Ethical implications of nudge interventions—whether from policymakers or the private sector—should be seriously considered from the outset of the conceptual design phase.

## Data Availability

The datasets presented in this article are not readily available because all raw data were deleted after completion of the study, which was originally a graduate thesis. However, sav. files will be shared upon reasonable request. Requests to access the datasets should be directed to marco.tagliabue@oslomet.no.

## Footnotes

1 Moderate violations are assessed based on the researcher’s interpretation of how strongly the trait of interest deviates from normality: for example, when data are not extremely nonnormal (Chi-squared, bimodal, or longtailed distributions; see 75).

2 We performed a nonparametric Mann–Whitney *U* test on the variable “commitment level of reducing screen time” following the failure to meet the normal distribution assumption in the active nudge condition, but this returned similar results to the t-test (*U* = 20, *p* = 0.109, Cohen’s *d* = 0.044–small).
