# Supplementary material for: Active nudging towards digital well-being: reducing excessive screen time on mobile phones and potential improvement for sleep quality
Source: Front Psychiatry. 2025 Jul 17;16:1602997. doi: 10.3389/fpsyt.2025.1602997 (PMC12310694; doi:10.3389/fpsyt.2025.1602997)
Supplement: Supplementary file 1 [file Supplementaryfile1.pdf]

Appendix

Scoring Range of 5-Point Likert Scale in the Survey

	Value	Range
Strongly Disagree	1	1.00 – 1.80
Disagree	2	1.81 – 2.60
Neither disagree nor agree	3	2.61 – 3.40
Agree	4	3.41 – 4.20
Strongly Agree	5	4.21 – 5.00

Scoring Range of 10-point Likert Scale in the Survey

	Value	Range
Not effective at all	1	1.00 – 1.90
Useless	2	1.91 – 2.80
Moderately useless	3	2.81 – 3.70
Slightly useless	4	3.71 – 4.60
Neither useless nor effective	5	4.61 – 5.50
Slightly effective	6	5.51 – 6.40
Moderately effective	7	6.41 – 7.30
Quite effective	8	7.31 – 8.20
Useful	9	8.21 – 9.10
Absolutely effective	10	9.11 – 10.00
